# Incoherence: A Generalized Measure of Complexity to Quantify Ensemble Divergence in Multi-Trial Experiments and Simulations

**DOI:** 10.3390/e26080683

**Published:** 2024-08-13

**Authors:** Timothy Davey

**Affiliations:** Research Department, London Interdisciplinary School, London E1 1EW, UK; tim.davey@lis.ac.uk

**Keywords:** ensemble, uncertainty, complexity, aleatoric, systems, replicability, ergodicity, indeterminacy

## Abstract

Complex systems pose significant challenges to traditional scientific and statistical methods due to their inherent unpredictability and resistance to simplification. Accurately detecting complex behavior and the uncertainty which comes with it is therefore essential. Using the context of previous studies, we introduce a new information-theoretic measure, termed “incoherence”. By using an adapted Jensen-Shannon Divergence across an ensemble of outcomes, we quantify the aleatoric uncertainty of the system. First we compared this measure to established statistical tests using both continuous and discrete data. Before demonstrating how incoherence can be applied to identify key characteristics of complex systems, including sensitivity to initial conditions, criticality, and response to perturbations.

## 1. Introduction

### 1.1. Motivation

The COVID-19 pandemic thrust *complexity science* into the public eye due to the emphasis on “following the science”. It demonstrated how the use of computational models to project the outcomes of policy decisions could be enormously powerful yet have immediate and real-world consequences for everyday lives. This influence and scrutiny are set to only increase as tough decisions need to be made in response to the worsening climate crisis.

Despite the field growing in prominence, there is no universally accepted definition of complexity. The word spans multiple disciplines, with a wide spread of informal definitions [1,2,3], leading to “the abuse of the terminology in non-scientific discourses” [4]. Where the ambiguity and misunderstanding is used as “a justification for indecision, suggesting that the science needs to mature before it can be helpful to decision-making” [5,6,7].

While a pragmatic approach would be to define complexity as “a collection of features rather than a single phenomenon” [8,9], quantifying these features remains a challenge. Analysts often rely on qualitative assessments or, in the absence of better options, fall back on standard statistical techniques. This is particularly worrying, as “studying the system via statistical mechanics would miss important properties” [2] brought about by the non-linear interactions, often leading to overconfidence and misleading interpretations. This limitation is well-recognized in fields like meteorology. However, other fields such as economics are still underestimating the importance of non-linearity [10,11,12,13].

These complex systems present global, cross-disciplinary challenges requiring urgent solutions. Therefore, we hope to join others in the effort [4] to clarify the meaning of these concepts with formal measures.

### 1.2. Aims

In this paper, we aim to prose a general measure that can detect and quantify whether a system is behaving in a complex way.

To do this, we will need to:

- offer an intuitive explanation for non-statisticians to understand this specific perspective of complexity (Section 3)
- determine how the baseline compares to other existing and commonly used metrics to quantify similarities and demonstrate its uniqueness (Section 5.1)
- explore how this measure behaves with the identified “features of complexity” to see if it can help to formally quantify and identify those features (Section 5.2)
- demonstrate whether the metric is actually valuable in real-world decision making (Section 6)

Since *complexity* is an extremely loaded and overused term, we will call this measure *incoherence*. As although this word has a clear meaning in the context of wave functions, this analogy fits well here.

## 2. Information Theoretical-Based Measures of Complexity

While numerous broad definitions of complexity exist, we focus on information theory as our foundation. This is because it has a well-developed and researched theoretical framework behind it; it has proven successful in many other areas of complexity science; and it offers a mathematically rigorous definition that is practical in real-world use cases [1,2,9].

That said, as emphasized in the introduction, although we need these measures to be formal, we also need them to be cross-disciplinary. Therefore, we aim to establish both intuitive and mathematical interpretations for each concept.

### 2.1. Shannon Entropy

Shannon formalized a measure called information entropy to help make messages over telecommunications more reliable [2]. Practically though, what the measure provides is a quantification for how much certainty a distribution offers. For instance, if the probability p(x) of the next letter in a message being *x* is 1, then this offers absolute certainty. Receiving that letter would give no extra *information*. If however, the letters were generated at random, then we would have maximum uncertainty, and by Shannon’s definition, maximum *information entropy*, as each new letter would provide maximum information. Visually, we can think of it as: The flatter the distribution, the more the probabilities are spread across the states; therefore, the greater the uncertainty, and thus, the greater the entropy.

Formally, information entropy *H* [14] for a discrete probability distribution *p* is given as follows:

(1) $$H\left(p\right)=\sum_{i}p\left(i\right)\frac{1}{\log p(i)}=-\sum_{i}^{n}p\left(i\right)\log p(i)$$

where for practicality concerns, if p(i)=0, the p(i)logp(i) term is considered to be 0. The units of *H* are *bits* if using $\log_{2}$ and *nats* if using the natural log. Throughout the rest of this paper, we shall use bits with $\log_{2}$.

The beauty of the entropy definition, however, is in its flexibility. Since it relies on probabilities, the definition itself can be adapted based on how we choose to define those probabilities. For example, when analyzing the frequency of edges (connections) in a network graph, they can be defined in multiple ways. In the simple case, we might define p(i) as the probability that a node has *i* edges, where p(i) is estimated by taking the frequency of nodes that have edges equal to *i* in a particular graph. However, in another study, it might be crucial that the nodes are distinguishable (i.e., they can be labeled). Here, we could estimate the probability $p_{j}\left(i\right)$ that node *j* has *i* edges by looking at an ensemble (multiple versions) of graphs containing *j* and taking the proportion of graphs where *j* has *i* edges.

The specifics of how we defined the probability distributions for each of the experiments in this paper are outlined in the Section 4.

### 2.2. Continuous Entropy

Similarly, since Shannon’s original definition was only for discrete distributions, others have since proposed various functions to estimate entropy for continuous distributions. The most commonly agreed upon technique uses a kernel density estimator (KDE) [15,16,17] and is the method we use in this paper.

This technique works by analyzing the distribution of observations within a defined range. It calculates the density *g* of observations around *R* evenly spaced points. A low variance in the set of densities *G* signifies a uniform distribution of observations across the range. In information theory, a uniform distribution corresponds to high entropy, reflecting the maximum uncertainty about the location of any given observation. Conversely, a high variance in *G* indicates that the observations cluster into specific regions, leading to a lower entropy and reduced uncertainty about their location.

More formally [18], we can define the density $g_{j}$ around reference point $r_{j}$ for *N* total observations *o* as follows:

(2) $$g_{j}=\frac{1}{N}\sum_{i}^{N}e^{-k∥r_{j}-o_{i}∥}$$

where when using R=100 reference points, the normalizing constant is set to k=100. We then convert this set of densities *G* into an entropy by normalizing the variance of $\hat{Var}\left(\sqrt{G}\right)$ by the variance of a maximum entropy distribution $V_{0}$ (a uniform distribution of observations) and the variance of the minimum entropy $V_{1}$ (where all observations are the same value). Finally, since there is an reverse relationship between this normalized variance and entropy, we subtract its value from 1, giving:

(3) $$H=1-\frac{\hat{Var}\left(\sqrt{G}\right)-V_{0}}{V_{1}-V_{0}}$$

where when using R=k=100, it has been calculated [18] that $V_{0}=0.0047683913076489465$ and $V_{1}=0.009711690148675985$. These values then ensure that *H* is bounded by [0,1].

### 2.3. Information Entropy as Complexity

One approach to defining complexity centers on the amount of information needed to describe a system [1,3]. Bar–Yann’s complexity metric [19] uses the information entropy required to describe a system at a specific scale.

Warren Weaver [20] offered a highly intuitive description of complexity. At one end of a scale, there are systems that are deterministic, where the causal relationships can be easily mapped out; for instance, how a few billiard balls might interact when hit together. In this case, Newtonian mechanics is a fantastic description of these systems. Then, as the system is scaled up to thousands of billiard balls, the individuals are lost, but the system as a whole is deterministic. Here, statistical mechanics work best. It is only when the causal relationships become more complex that the system becomes complex.

This is why defining complexity as simple entropy has faced significant criticism [2,4]. High-entropy systems are normally only obtained if the behavior is statistical, meaning that entropy on its own is poorly correlated with complexity.

### 2.4. Gershenson–Fernandez Complexity

Recognizing this, there are several other measures that sought to cast complexity as a balance between order and disorder. Examples include effective complexity [21], Langton’s λ [22], Wuensche’s *Z* [23], and Lopez–Ruiz complexity [24].

In an effort to consolidate and reduce confusion, Gershenson and Fernandez proposed [4] a unified, formal definition $C_{GF}$ along the same lines:

(4) $$C_{GF}=aI\left(1-I\right)=4\frac{H}{H_{max}}\left(1-\frac{H}{H_{max}}\right)$$

This measure is bounded by [0,1], as the entropy *H* is normalized by the theoretical maximum $H_{max}$ for the given number of states. $C_{GF}$ has the notable property that when *H* is at small or large extremes, so is $C_{GF}$. Instead, the maximum occurs when the system is most balanced at $H/H_{max}=0.5$.

This is a strikingly refined definition of complexity, particularly as it is derived from their equally elegant definitions of emergence and self-organization.

Intuitively, we can test this definition using Sean Carroll’s thought experiment (https://youtu.be/MTFY0H4EZx4, accessed on 4 June 2023): “Take a cup filled with half coffee and half milk. It starts off in a state with relatively low entropy…It’s a simple setup, milk on top, coffee on the bottom. Now, as the milk and coffee begin to mix, entropy goes up where they are mixed together…But the system also becomes more complex to describe what you see, you would have to specify exactly how all of those tendrils of milk and coffee intricately swirl into each other. Continuing on, entropy keeps going up…this highly-mixed equilibrium is once again simple, it’s just a homogenous mixture”.

Here $C_{GF}$ certainly does a fantastic job at identifying the middle region. But what if instead of a cup of coffee we looked at an ideal gas expanding from a small box into a larger one? This, too, would go through a similar low to high entropy transition.

You could certainly define the central region of a gas as being complex, because it is rapidly changing and would require additional parameters to describe the system. However, as we have seen, many definitions of complexity revolve around the presence of non-linear casual relationships. For instance, Gershenson and Fernandez write [4], “Etymologically, the term complexity comes from the Latin *plexus*, which means interwoven. In other words, something complex is difficult to separate“. By definition, the molecules of an ideal gas do not interact and are entirely separate, even during the transition period. On the other hand, the fluid dynamics involved with mixing a coffee are very much non-linear and complex.

Therefore, the question we ask here is: can we distinguish the middle region from the extremes, but now only for interwoven systems?

## 3. Defining Ensemble Complexity

### 3.1. Intuitive Description

The field of thermodynamics introduced the concept of an *ensemble*, where for a set of macroscopic, measurable conditions (like pressure and volume), there is an ensemble of possible micro-states that the system can be in. Each micro-state represents a unique value of immeasurable conditions (like the velocity and position of each particle) that still give rise to the same macro parameters. Each of these micro-states can be interpreted as either multiple experimental trials under the same macro-conditions or the many different paths that a single experiment could have taken.

If we were to inspect an ensemble of coffees, they would all start in the *exact* same low-entropy state with the milk on top and coffee on the bottom. Then, while they were being mixed, we would see clearly identifiable and unique features such as the formation of swirls. These self-organized patterns would be of a relatively lower entropy and are yet another common signal of complexity [2]. Finally, all the cups would end in the *exact* same high-entropy equilibrium state.

However, if we semi-transparently layer each coffee on top of each other so that the observations are *pooled* into a single observation, those semi-ordered swirls would now be far less identifiable. The central region would now appear to be much more mixed and so have a higher entropy.

Therefore, by measuring the difference in entropy between the pooled cup and individual ones, we should be able to provide a measure of complexity.

This method also helps us tell the difference between the coffee and the ideal gas examples, as in every instance, the gas would expand in a near identical fashion, where each would fill the available space in a sphere at a uniform rate. This means that the pooled system would also have an identical entropy to the individual ones; therefore no complexity would be measured.

### 3.2. Types of Ensemble Uncertainty

At this point, it is important to become more rigorous about which types of uncertainty incoherence will be measuring. In broad terms, there are two main branches of uncertainty [25,26].

1. Epistemic (sometimes called ontological), which arises due to a lack of knowledge about a system. This mean that the uncertainty can be reduced by collecting more data; for instance, by tossing a coin thousands of times to determine if it is biased. Within this category, though there is:
  (a) Structural (or model misspecification), which describes how well the equations and model actually reflect the real-world dynamics.
  (b) Parametric, which reflects how well you are able to estimate your models parameters.
2. Aleatoric (called internal variability in climate science or endogenous in economics), on the other hand, cannot be reduced by collecting additional data. It arises due to the very nature of the system being non-deterministic and stochastic. For instance, even if you know a coin is biased toward heads by 80% in the aggregate, each time you actually flip the coin, the result will always have some level of uncertainty.

We expect our measure to be able to quantify both parametric and structural uncertainty by analyzing ensembles that have varied their macro parameters or the models used. But in our case, it is crucial we are able to isolate and then quantify only the aleatoric uncertainty, as this is explicitly where complex dynamics manifest.

### 3.3. Existing Commonly Used Ensemble Measures

The use of ensembles was pioneered in meteorology [27,28,29,30], where it was recognized by Ed Lorenz that the Naiver–Stokes equations being used were chaotic and sensitive to initial conditions. However, Tim Palmer [10] then recognized that if the predictions of an ensemble were combined, the aggregate prediction was more accurate than any individual one (analogous to Sir Francis Galton’s wisdom of crowds effect [31]), where understanding the confidence in the predictions of these highly chaotic systems became as important as the prediction itself. Some of the most commonly used methods are as follows:

- The root mean square error (RMSE). Here, we compare the predicted value against a real-world historic truth. This measure is extremely useful when trying to reduce structural uncertainty through model improvement. However, it cannot be used when analyzing projections into the future where we have no truth to compare [32].
- The mean of the pooled distribution (or mean of means). This a very pragmatic measure when we need to be able to provide a single number to act upon, particularly for systems that have a high parametric but low aleatoric uncertainty. However, as we will see later in Figures 17 and 19, these measures can be misleading when the system is non-ergodic. For instance, a coin cannot be in both a state of heads and tails.
- Proportion or percentage is often used when dealing with these scenario-based questions. For instance, in weather forecasts, the public is used to an X% rain prediction, which is simply the proportion of trials where rain was recorded in the ensemble. This is an extremely effective and highly intuitive; however, it does require we first know what scenarios we are looking for and then be able to articulate them as a binary outcome. In this paper, we are actively aiming to create a measure to be used upstream of this, which can detect variations in output so that we can efficiently invest our efforts.
- Brier scores are then used to calculate the accuracy of the percentage predictions against reality. Like RMSE, we do not compare these measures in this paper, as we are focused on the aleatoric uncertainty of projections.

### 3.4. The Use of Statistical Tests

Statistical tests are another common method used to compare distributions. With these, one starts with a *null hypothesis* of there being no relationship or no difference between the distributions. The tests are then used to precisely determine whether a set of observations falls outside of what is expected by that null hypothesis. In our case, they assume that a system is not complex (i.e., there is no difference in the ensemble). Then given enough evidence, one can then say that the system is complex. This makes these tests non-ideal for our use case, firstly, because statistical tests are explicitly about the epistemic uncertainty around how much data one has. Secondly, the burden of proof is on demonstrating that a difference exists, which is exactly what we want if we were testing whether a drug is effective, but the opposite assumption should be made when dealing with complex dynamics.

There are a vast number of statistical tests available depending on the data [33]. Each makes different assumptions or requires specific data formats. For instance, there are different tests for continuous and discrete data; many tests assume the distributions are Guassian; that there is no differences in the variance of the distributions; or there are only two distributions being compared at once. This makes it even more difficult for non-expert stakeholders to engage with the data. That said, for completeness, we aim to compare the appropriate tests so we can given their limitations in Section 5.

### 3.5. Conditions of a New Measure

Before selecting or proposing the new measure, it is important to clearly lay out the conditions that is should satisfy.

- The measure should be continuous, consistent, and bounded by [0,1]. There are many measures (such as standard deviation σ) that, although are useful, are also incredibly sensitive to scale. For instance, a normal distribution of μ=1,σ=1 is very different from another with μ=1000,σ=1. This means that in order to be able to fully understand measures such as σ, we need the full context. This means it is extremely hard to compare σ across different systems; therefore, it cannot be used to detect complex dynamics at a large scale. Therefore, it is vitally important that our measure be bounded by [0,1].
- The measure is minimal at 0 when each of the distributions in the ensemble is identical. This is to ensure that we can be confident that the results are consistent with each other and there are no complex or chaotic dynamics that are affecting the output of the model.
- The measure is maximal at 1 when given an identity matrix where the identity matrix is defined as $I_{n}=diag\left(1,1,1,1\right)$, ${\left(I_{n}\right)}_{ij}=\delta_{ij}$ so that $I_{1}=\left[1\right],I_{2}=\left[\left[1,0\right],\left[0,1\right]\right]$. This is because the identity matrix represents the highest level of dissonance, as each individual distribution of the identity matrix represents the maximum possible certainty (and therefore the lowest entropy). Meanwhile, the ensemble as a whole represents the maximum *uncertainty*, with the pooled distribution being in its highest-entropy state.
- The measure should be compatible with both discrete and continuous data. For the same reason as (i), we want incoherence to be used to compare across systems. In general, when analyzing systems, we tend to have to pick the measure to fit the data. However, this makes it extremely hard to then have meaningful conversations with stakeholders who are likely unfamiliar with these many unique measures.
- The measure is invariant to the total number of distributions, and in the discrete case, it is invariant to the total number of states. We want this measure to be of the aleatoric uncertainty of the system, not the epistemic uncertainty of the data.
- The measure is consistent across systems and is not subject to the overall average entropy of the system. We want incoherence to be as interpretable and consistent as possible, meaning we want to ensure that systems with a higher baseline entropy do not have higher incoherence values. If anything, it makes more sense that any inconsistency in the results of more ordered systems (with low entropy) should be weighted more highly, since we would expect more ordered systems to stay ordered across trials.
- The measure is pessimistic and errs on the side of assuming the presence of complex dynamics, with an emphasis on detecting outliers. The downside of treating a coherent system as complex is much smaller than the downside of treating an incoherent system as statistical. An example of this is a black swan, which is often a consequence of complexity and long-tail distributions. In these situations, a single, extremely rare (and unpredictable) event can be catastrophic for the system as a whole. Having an emphasis on these outlier distributions is vital for a practical complexity measure.

### 3.6. Definition of the Pooled Distribution

Going back to our intuitive description above, the question becomes, what is the best way to compare the difference between the pooled distribution and a single trial?

Therefore, to specify the pooled distribution $\bar{P}=\left\{p\left(0\right),p\left(1\right),...p\left(i\right)\right\}$, we propose that is it is simply the mean of the *K* probability distributions $\left[P_{1},P_{2},...P_{K}\right]$ with states *i* contained in the ensemble, such that:

(5) $$\bar{P}\left(i\right)=\frac{1}{K}\sum_{k=0}^{K}p_{k}\left(i\right)$$

where using (1), we can find the Shannon entropy of the pooled distribution $\tilde{H}=H\left(\bar{P}\right)$. Intuitively, this is the same as combining the observations from all distributions into a single distribution, then normalizing it into a probability distribution.

It should be noted that (5) is specifically used for the discrete case. However, the same method works for the continuous case, where we combine the observations from all distributions into a single set of observations, then find the continuous entropy, now using Equation (3).

### 3.7. Choosing a Divergence Metric

#### 3.7.1. f-Divergences

Next, the most common way to quantify the difference between two probability distributions is using a class of function known as f-Divergences [34]. Examples include the total variation distance, Pearson divergence, and Bhattacharyya distance, to name a few. The most commonly used are the Kullback–Leibler divergence $D_{KL}$ and Hellinger distance $D_{H}$.

These are defined as follows for discrete probability distributions *P* and *Q*. In our case we treat *P* as the individual trial and *Q* as the pooled probability distribution $\bar{P}$.

(6) $$D_{KL}\left(P||Q\right)=\sum P\left(i\right)\log\left(\frac{P(i)}{Q(i)}\right)$$

(7) $$D_{H}\left(P,Q\right)=\frac{1}{\sqrt{2}}\left|\right|\sqrt{P}-\sqrt{Q}{\left|\right|}_{2}=\frac{1}{\sqrt{2}}\sqrt{\sum_{i}{\left(\sqrt{p(i)}-\sqrt{q(i)}\right)}^{2}}$$

Unfortunately, $D_{KL}$ and $D_{H}$ are only defined for discrete distributions. Also, they do not meet a number of the conditions (Section 3.5) outlined above, including not being bounded. That said, we analyze and compare these measures in more detail in Section 5.

#### 3.7.2. Jensen–Shannon Divergence

For our purposes, the most promising f-divergence is the Jensen–Shannon divergence $D_{JS}$ [35]. It is a symmetrized and smoothed version of $D_{KL}$. For a ensemble of probability distributions $\left[P_{0},..P_{K}\right]$, it is defined as follows:

(8) $$D_{JS}=H\left(\sum_{k}w_{k}P_{k}\right)-\sum_{k}w_{k}H\left(P_{k}\right)$$

where when equal weights are used $w_{k}$, the first term in (8) is the entropy *H* of what we have defined as the pooled distribution $\bar{P}$ (5), making $D_{JS}$ the weighted average of the difference between $H\left(\bar{P}\right)=\tilde{H}$ and the entropies of the individual distributions $H_{k}$. Therefore, with uniform weights $w_{k}=1/K$, we can expressed it as follows:

$$D_{JS}=H\left(\bar{P}\right)-\sum_{k}\frac{H(P_{k})}{K}=\frac{1}{K}\sum_{k}\left(\tilde{H}-H_{k}\right)$$

What is extraordinary about this measure is how closely it informally matches the intuitive description outlined above. But what makes it particularly promising is that it is generalized beyond just two distributions. It does this by not directly comparing the distributions but also comparing them to the pooled distribution, where the pooled distribution can be composed of any number of individuals. Additionally, since it compares the entropies rather than probability distributions, it can be used with both continuous and discrete data (or indeed any more nuanced chosen entropy estimator).

Unfortunately, like $D_{KL}$ and $D_{H}$, it has shortcomings, which are explored in Section 5. That said, given its benefits, it can be used as a solid foundation to build our measure around.

#### 3.7.3. Earths Mover’s Distance

Another popular measure (which is not an f-divergence) is the Earth mover’s distance [36], sometimes known as the Wasserstein metric $D_{EM}$. Intuitively, this can be thought of as the minimum cost of building a specific smaller pile using dirt taken from a given larger pile (where the pile of earth is an analogy for probability distributions).

**Figure 1:**
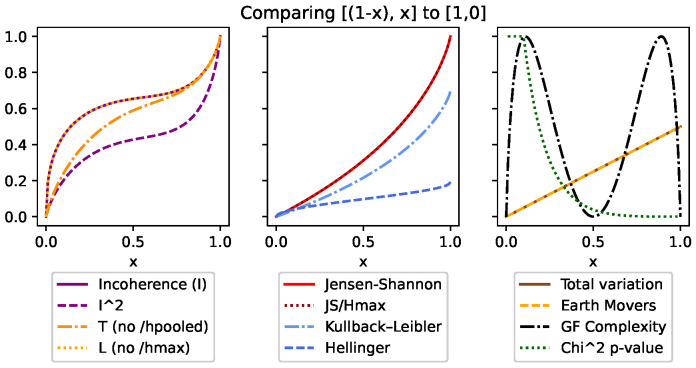
Here, we compare [[(1−x),x],[1,0]] to demonstrate the shape of these key measures across a full range of difference. The first thing to note is that the variants of incoherence and Jensen–Shannon divergence are the only measures that are normalized within these extremes. We can also see based on the difference in incoherence and T (11) how dividing by $\tilde{H}$ affects the lower values.

**Figure 2:**
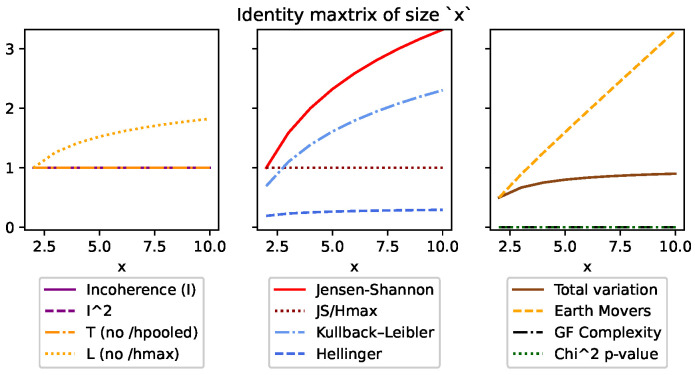
This figure looks at an identity matrix of size *x*. Incoherence and the normalized Jensen–Shannon divergence ($JS/H_{max}$) are the only measures to be consistent and bounded at 1.

**Figure 3:**
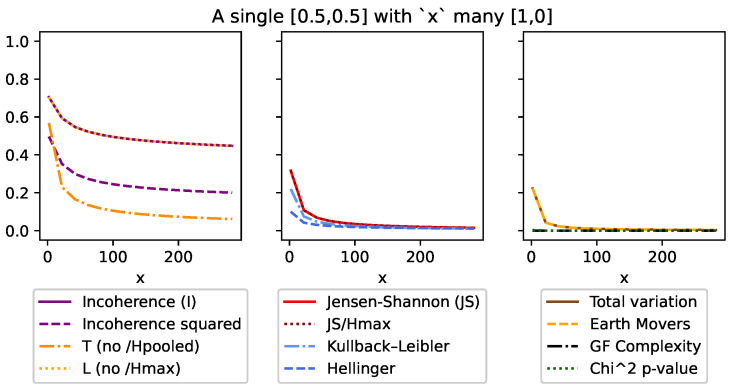
This figure compares a single high-entropy distribution [0.5,0.5] with *x* low-entropy [1,0] distributions. What we see is incoherence (and all measures) reduce as the ensemble becomes more homogeneous. But, we should also note how incoherence reduces much more slowly as it emphasizes outliers more. This is in part due to squaring the divergence (known as taking the second moment) and dividing by $\tilde{H}$ (as can be seen compared to *T* (11)).

**Figure 4:**
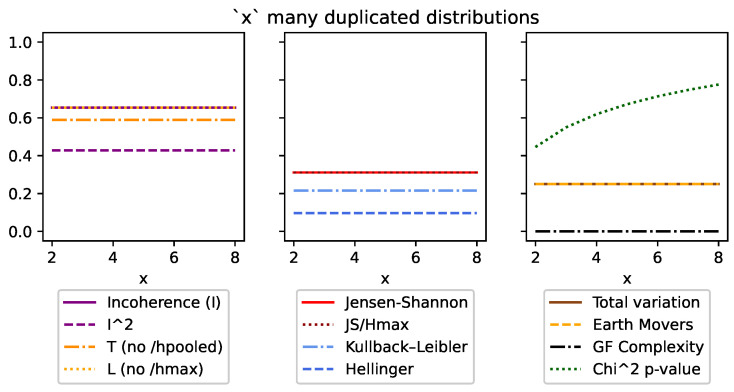
This figure looks at *x*-many duplicated highest-entropy and lowest-entropy distributions. The first thing to note is how all the divergences are consistent to the additional distributions, which is to be expected. The only exception to this is the $\chi^{2}$ value, which counter-intuitively increases (i.e., becomes less confident) with more distributions. This is because more distributions require more data in the $\chi^{2}$ interpretation.

Although $D_{EM}$ can also be used with both continuous and discrete distributions, like all of the other divergences, it is an absolute measure and unfortunately does not meet many of the same conditions (Section 3.5). This can be seen in Figure 1, Figure 2, Figure 3, Figure 4, Figure 7 and Figure 8, and it is explored more in the results.

### 3.8. Definition of Incoherence

Building upon $D_{JS}$, we propose incoherence *I* to be as follows:

(9) $$I^{2}=\frac{1}{K\tilde{H}H_{max}}\sum_{k=0}^{K}{\left(\tilde{H}-H_{k}\right)}^{2}$$

where $H_{max}$ is the maximum possible entropy for the given number of states *i*. In the discrete case, this can be found in (10) to be $H_{max}=\log_{2}\left(i\right)$ [14,37]. In the continuous case (3), it is defined as $H_{max}=1$, as it is bounded by [0,1] [18].

Incoherence is lower-bounded by I=0 when the probability distributions $P_{k}$ are identical, $P_{k}=P_{1}=P_{2}$. This is because using (5), we can find the pooled distribution to be identical to any of the individual ones $\bar{P}=P_{k}$, meaning the entropies are also identical $\tilde{H}=H_{k}$. This leads to each term of the summation in (9) to be $\tilde{H}-H_{k}=0$, and therefore, I=0.

Incoherence is then upper-bounded by I=1 for an identity matrix of any size. For instance, taking an identity matrix of size 2, which is defined as $\left[\begin{array}{cc} 1 & 0\\ 0 & 1 \end{array}\right]$, the Shannon entropy (1) of any row would be as follows:

$$H_{k}=-\sum p_{i}\log_{2}\left(p_{i}\right)=-1\times \log_{2}\left(1\right)-0\times \log_{2}\left(0\right)=-1\times 0-0=0$$

This is true for an identity matrix of any size, as the singular p(i)=1 term is $\log_{2}\left(1\right)=0$ and the N−1 other p(i)=0 terms are defined as $0\times \log_{2}\left(0\right)=0$.

The pooled distribution $\bar{P}$ (5) is then given by the following:

$$\bar{P}=\frac{1}{N}\left[1_{0},1_{1},...1_{N}\right]=\frac{1}{2}\left[1,1\right]$$

This is a uniform distribution, which Shannon defined as being the maximum entropy. For our 2 state identity matrix, we then have the following:

$$\tilde{H}=-\sum^{N}\frac{1}{N}\log_{2}\left(\frac{1}{N}\right)=-\frac{1}{2}\log_{2}\left(\frac{1}{2}\right)-\frac{1}{2}\log_{2}\left(\frac{1}{2}\right)=-2\times \frac{1}{2}\times -1=1$$

when given a uniform probability distribution $H=H_{max}$ [37], because:

(10) $$H_{max}=-\sum^{N}\frac{1}{N}\log_{2}\left(\frac{1}{N}\right)=\frac{N}{N}\log_{2}\left(N\right)=\log_{2}\left(N\right)$$

for the identity matrix $\tilde{H}=H_{max}$. Finally, since *I* is maximal when the $\tilde{H}-H_{k}$ term is maximal, and $H_{k}=0$, we have the following:

$$I_{max}=\sum^{K}\frac{{\left(H_{max}-0\right)}^{2}}{KH_{max}H_{max}}=K\frac{H_{max}^{2}}{KH_{max}^{2}}=1$$

It should be noted that $H_{k}$ can be greater than $\tilde{H}$ in some cases. For example, if we had an ensemble of one high-entropy distribution $P_{h}=\left[\frac{1}{2},\frac{1}{2}\right]$ alongside *N* low-entropy distributions $P_{l}=\left[1,0\right]$, then the pooled distribution would be $\bar{P}=\left[\frac{N}{2},\frac{1}{2}\right]$, meaning as *N* increases, $\bar{P}⟶P_{l}$, causing the pooled entropy to be less than the maximum entropy distribution $\tilde{H}<H\left(P_{h}\right)$. But, even as N⟶∞, the pooled entropy would still contain some $P_{l}$ and would approach a small but positive value of α. From an intuitive perspective, as the ensemble becomes more consistent, incoherence and $D_{JS}$ reduce toward 0 (from a maximum value of I=0.654). This can also been seen as follows:

$$\lim_{N\to \infty }\frac{1}{N\tilde{H}H_{max}}\left({\left(\tilde{H}-H_{max}\right)}^{2}+\sum^{\left(N-1\right)}{\left(\tilde{H}-0\right)}^{2}\right)=\frac{1}{N\alpha H_{max}}\left({\left(\alpha -H_{max}\right)}^{2}+\left(N-1\right)\alpha^{2}\right)=0$$

This specific case can be seen in Figure 3 where 2<x=N<300.

These boundaries satisfy the conditions (Section 3.5) (i, ii, iii) laid out above. Condition (iv) is also satisfied because we can use the discrete (1) and continuous (3) entropy estimators rather than the raw probability distributions.

Condition (v) first requires that incoherence is consistent, no matter the number of distributions provided. This is a common requirement, and it is met by many of the divergences by taking the weighted sum. In our case, to ensure the measure is also bounded by 1, we used the *mean* divergence by setting the weights to $w_{k}=1/K$.

**Figure 5:**
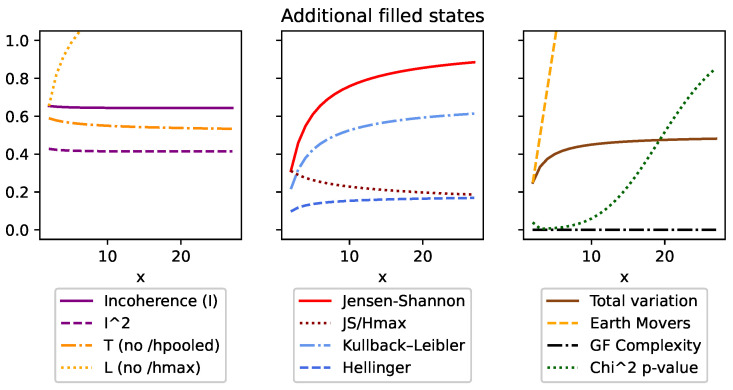
This figure compares one minimum entropy to one maximum entropy distribution, each of length *x*, so that when x=3, we have [[1,1,1],[1,0,0]]. In this case, incoherence is the only measure that sees this comparison as equivalent and so is invariant.

Secondly, condition (v) requires that the measure is invariant to the total number of states in discrete distributions. We have compensated for this by ensuring the upper bound of 1 was consistent no matter how many states the distributions had. Since the maximum value of $D_{JS}=H_{max}$, we can normalize $D_{JS}$ and *I* by simply dividing by $H_{max}$. This is demonstrated in more detail in the results and can be seen in Figure 2 and Figure 5.

Condition (vi) requires that systems with a lower average entropy (i.e., ones that are more ordered) have a higher incoherence. We chose to do this by dividing the divergence by the pooled entropy $\tilde{H}$. To illustrate, let us compare a small deviation in a high-entropy system against a low-entropy system:
**Distributions**$\tilde{H}$$D_{\mathrm{JS}}$$D_{\mathrm{JS}}/\tilde{H}$I[0.9,0.1,0.0,0.0,0.0],[0.8,0.2,0.0,0.0,0.0]0.60.0140.0240.107[0.2,0.2,0.2,0.2,0.2],[0.4,0.0,0.2,0.2,0.2]2.20.1250.0550.103

We see here that there is a 10× difference in the $D_{JS}$ values. This is because $D_{JS}$ is an absolute measure, and differences in higher-entropy systems are measured to have higher divergences. We want these divergences to be relative to the system. In the case above, we see the $D_{JS}/\tilde{H}$ values now only have a 2× difference.

Since we have already normalized by $H_{max}$, we must take the square of divergences to be able to also normalize by $\tilde{H}$. Again, this is investigated in more detail in the results. Figure 1 shows how incoherence is boosted at lower values, but more importantly, Figure 13 shows how it keeps incoherence invariant.

Finally, condition (vii) requires a bias toward over-weighting small changes and outliers. This is achieved in two ways. First, by squaring the divergence for condition (vi), the measure becomes analogous to the standard deviation. By using the *second moment*, the focus is now on the variance in divergences rather than the expected value of the divergences. The square makes the values absolute and places a greater importance on higher divergences, making even small outliers more prominent. This can be seen in Figure 3. Similarly, because the summation is bounded by [0,1], taking the square root further increases the pessimism at lower values.

## 4. Methods

The proposed measure is analyzed in two parts: first, how it compares to other standard measures, and second, the purpose of this metric is to explicitly identify so-called features of complexity. Therefore, we shall explore how this measure behaves when these features are present. However, it is important to note that since complexity has no clear verbal definition (let alone a mathematical one), these explorations lack the ability to be quantitatively verified as correct. Therefore, to compensate for this, we shall compare it to well-studied models and features within the complexity community where a broad consensus has been reached. This will then further demonstrate the usefulness of the metric by offering a consistent quantitative metric for these cases.

### 4.1. Baseline with Existing Metrics

#### 4.1.1. Discrete Data

Here, we aim to understand the behavior of incoherence *I* (9). We do this by looking at its shape for six specific cases that are designed to check the conditions (Section 3.5) outlined above. For completeness, it is also compared to the alternative measures considered, specifically Jensen–Shannon divergence $D_{JS}$ (8), a normalized version of Jensen–Shannon divergence $D_{JS}/H_{max}$, total variation $D_{TV}$, Earth mover’s distance $D_{EM}$, Kullback–Leibler divergence $D_{KL}$, Hellinger distance $D_{H}$, the *p*-value of the $\chi^{2}$ metric, and the maximum $C_{GF}$ (4) recorded for any of the distributions in the ensemble, as well as an incoherence alternative *T* where it is not normalized by $\tilde{H}$ (11).

(11) $$T=I\sqrt{\frac{\tilde{H}}{H_{max}}}=\sqrt{\sum_{k}^{K}\frac{{\left(\tilde{H}-H_{k}\right)}^{2}}{KH_{max}^{2}}}$$

(12) $$L=I\sqrt{H_{max}}=\sqrt{\sum_{k}^{K}\frac{{\left(\tilde{H}-H_{k}\right)}^{2}}{K\tilde{H}}}$$

Finally, because $\chi^{2}$ uses absolute values rather than probability distributions, each of the described cases was multiplied by 10 before the $\chi^{2}$ *p*-value was calculated.

#### 4.1.2. Continuous Data

For another six specific cases, we used the Python package NumPy to generate *N* random continuous distributions. As above, the specifics of each case are described alongside the result.

Since the distributions were generated randomly, we ran each trial M=50 times. The mean of each measure was then plotted using the standard deviation as error bars.

Here, incoherence *I* (9) was compared alongside the standard deviation of the means σ(μ), the standard deviation of standard deviations σ(σ), $D_{JS}$ (8), the maximum $C_{GF}$ (4), Earth mover’s distance, ANOVA *p*-value, and Kruskal *p*-value.

### 4.2. Features of Complexity

As mentioned, to investigate the value of incoherence when features of complexity are present, it was important to test it against a variety of well-studied models. The code for the simulations and analysis is open-source and is available to develop further (https://github.com/timjdavey/Incoherence, accessed on 4 June 2024).

#### 4.2.1. Erdos–Renyi Graphs

First, we looked at Erdos–Renyi Graphs [38] (ER-Graph). These have been well studied [9] in the context of complexity [4]. They are a graph *G* with *n* nodes, where each edge between two distinguishable (labeled) nodes is created randomly with a probability of *p*, independently from any other edge.

To investigate the systemic uncertainty of the graphs, we created a slightly modified version of this algorithm. Each edge is now created randomly with a probability of p(1−q)+rq, where 0.0<r<1.0 is a random number defined on a per-graph basis. When q=0, the system behaves like a standard ER graph, but when q=1, the parameter *p* is effectively meaningless.

The entropy of these graphs is estimated by creating a distribution p(i) of edge counts, i.e., the number of nodes with *i* edges, where the pooled distribution follows (5).

#### 4.2.2. Cellular Automata

Next, we looked at 1D cellular automata (CA) consisting of *n* cells evolved over *t* steps. The well-documented 256 Wolfram rules [39] were used as proxies to simulate different system dynamics.

The information entropy (1) for each sample was approximated using the standard approach of the cellpylib (https://github.com/lantunes/cellpylib, accessed on 4 June 2024) library (used to generate the CA), where $p_{a,k,i}$ was calculated using the frequency of its boolean states *i* over time for a given cell *a* in a particular sample *k*. However, we used the stable entropy [4] of the system, which only uses the second half ((T/2:T]) of the observations (to allow the CA to stabilize). We also chose to calculate the diagonal entropy (https://demonstrations.wolfram.com/CellularAutomataOrderedByEntropy/, accessed on 4 June 2024), as stable diagonal patterns are a common feature in CA. The different entropy measures and their corresponding incoherence values were calculated independently of each other. The initial state of the CA was the varied micro-variable across samples.

#### 4.2.3. Daisyworld

Developed to investigate the *Gaia hypothesis* [40], the standard version of the Daisyworld model was ported from the heavily studied Netlogo version (https://ccl.northwestern.edu/netlogo/models/Daisyworld, accessed on 4 June 2024). The model is set up so that there is a world made up of $X^{2}$ cell grids. The model has a sun, which shines on the world evenly at a luminosity *l*.

The world is populated by species of daisies. Each species has a specific albedo *a*, where a higher *a* value reflects light away from the planet, thus cooling the local area. The daisies can only survive within a set temperature range. If they do survive, they randomly populate into neighboring cells. They also have a maximum age, after which that specific daisy dies. This dynamic, under certain conditions, often causes the global temperature (the average of the local cell temperatures) to stabilize under certain conditions, as the daisies breed to an optimum level for their survival.

The information entropy for this model was calculated using the continuous distribution of temperatures. The initial condition varied across samples was the positions of the daises. The absolute initial number of each species, the world size, albedos, and solar luminosity were kept consistent across samples (similar to the CA models).

Again, we expanded this standard model in three ways: first, so that the world could be populated by any number of species each with their own albedo; second, including the ability for daisies to mutate, where each time a daisy seeds, there is a probability that their offspring mutates to have a slightly different albedo from their parents by a small random amount. Third, the model introduced the ability to perturb the system by causing a plague, where at a given time *t*, a certain proportion *r* of randomly selected daises from species *s* would die.

## 5. Results

### 5.1. Baseline with Existing Metrics

#### 5.1.1. Discrete Data

We started with top row of Figure 1 by looking at the overall shape made over the extremes. This was done using a distribution [[1,0],[x,(1−x)]] such that when x=0, we have identical distributions, [[1,0],[1,0]], and when x=1, we have entirely opposite distributions, [[1,0],[0,1]]. The most interesting thing to note is that *I*, *T*, and $D_{JS}$ are the only measures to trace between the [0,1] bounds. That said, $C_{GF}$ displays its characteristic shape, as it follows the changes of the second distribution. But, because of the symmetric nature that distribution, *C* contains two peaks. Finally, it is worth noting the difference between *I* and *T* for the lower values. This is an artifact of that fact that $\tilde{H}$ is lower when the distributions are more similar. This is seen as a positive, as we want *I* to be pessimistic and measurably higher, even for small inconsistencies between distributions.

Figure 2 looks at the behavior of the measures when given an identity matrix of size *x*, so that when x=3, we are comparing [[1,0,0],[0,1,0],[0,0,1]]. This distribution represents the most inconsistent results possible, as not only is each row different from every other row, but they are all in the most ordered state possible and cover all possible outcomes. Here, we see *I* and $D_{JS}/H_{max}$ are the only values that stay consistent at the 1 upper bound. The others are all absolute measures, which increase as the maximum entropy of the distributions increase.

In Figure 3, we look at one maximum entropy distribution $P_{h}=\left[\frac{1}{2},\frac{1}{2}\right]$ alongside *x* minimum entropy distributions $P_{l}=\left[1,0\right]$. Here, all the measurements follow intuition by dropping as the ensemble becomes more consistent. That said, it is worth noting that *I* drops at a slower rate than *T* because as an ensemble becomes more consistent as the $\tilde{H}$ value also drops. We see this side effect as a positive, as it helps *I* to more strongly identify outliers.

Figure 4 looks at an ensemble of *x* many $\left[\frac{1}{2},\frac{1}{2}\right],\left[1,0\right]$ duplicated distributions, so that when x=2, the ensemble is $\left[\left[\frac{1}{2},\frac{1}{2}\right],\left[1,0\right],\left[\frac{1}{2},\frac{1}{2}\right],\left[1,0\right]\right]$. In this case, all the divergence measures are invariant. The only outlier is $\chi^{2}$, which increases because the overall number of observations is increasing. However, during the translation to the *p*-value, the confidence decreases (and the *p*-value increases). This is because it considers additional distributions as a degree of freedom and therefore requires more data per distribution to increase confidence.

Figure 5 looks at the impact of additional states. Here, we compare $\left[\left[1_{0},1_{1}...1_{x}\right],\left[1_{0},0_{1},...0_{x}\right]\right]$, where for x=3, we have $\left[\left[\frac{1}{3},\frac{1}{3},\frac{1}{3}\right],\left[1,0,0\right]\right]$. This means that no matter the value of *x*, we are always comparing the highest-entropy state to the lowest. Here, we see incoherence is the only measure that is invariant.

**Figure 6:**
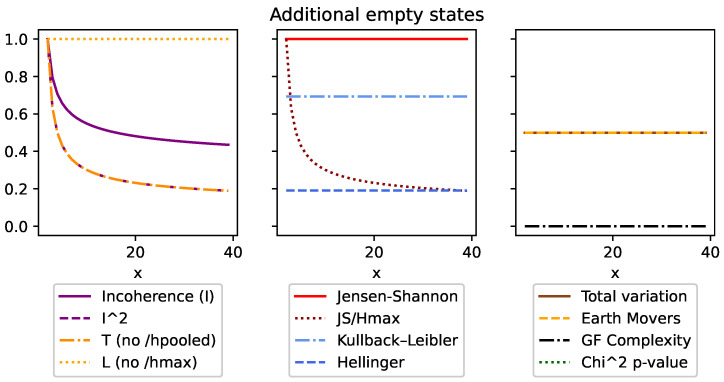
This figure compares two minimum entropy distributions of length *x*, such that when x=3, we have [[1,0,0],[0,1,0]], and x=4 gives [[1,0,0,0],[0,1,0,0]]. Here, incoherence decreases. Unlike Jensen–Shannon divergence, it sees the consistency of the additional zeros as a measure of coherence.

Figure 6 looks at an ensemble of $\left[\left[1_{0},0_{1}...0_{x}\right],\left[0_{0},1_{1},...0_{x}\right]\right]$, where if x=4, we have [[1,0,0,0],[0,1,0,0]]. In this case, both distributions are inconsistent with each other and are also at their lowest entropy. Here, we see that many of the divergence values are now invariant because they work with absolute measures and therefore discard the additional zero states. Incoherence, on the other hand, decreases, as although there is no overlap of their probabilities, there is increasing agreement about where their distributions are not. Intuitively, we can understand this as the similar zero states count toward the ensembles consistency. Meanwhile, $\chi^{2}$ for the top row decreases in confidence with additional states, as more states means more degrees of freedom, which requires a greater burden of proof. However, this perspective is inconsistent, as in the bottom row, even with the presence of additional states, the $\chi^{2}$ measure ignores them because they are zero across the entire ensemble.

#### 5.1.2. Continuous Data

**Figure 7:**
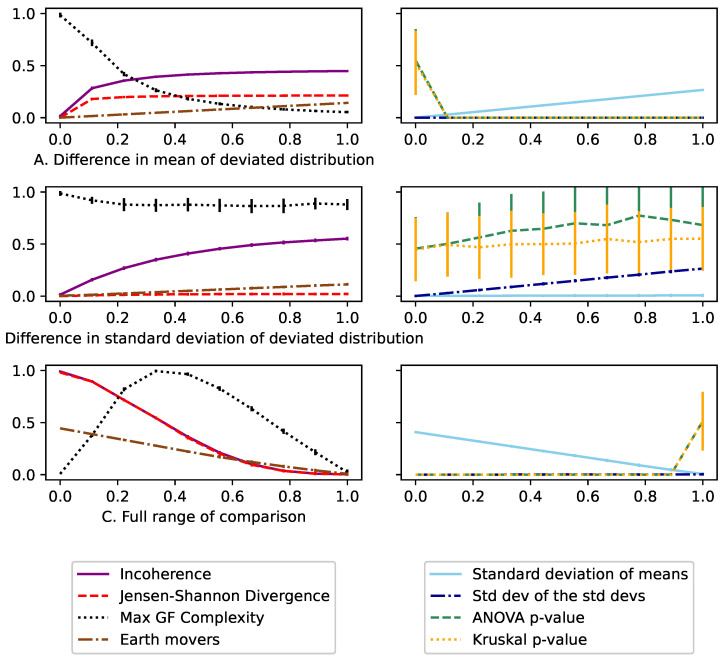
The **top row** shows how all standard measures are good at detecting a difference in μ of multiple Guassian distributions. The **middle row**, however, shows that only incoherence and the standard deviation of standard deviations σ(σ) can detect a difference in σ. Even then, the σ(σ) value only works in this case because it is comparative, as the value is relative to the change; therefore, it is hard to obtain a baseline using real-world data. Meanwhile, the (**bottom**) graph shows how incoherence behaves between its bounds [0,1].

The top row (A) of Figure 7 is composed of 12 Guassian distributions with μ=1 and σ=0.01, plus a single distribution with μ=1+x and σ=0.01. Here, we see that all the analyzed measures are effective at detecting this change in mean. However, the *p*-values offer no ability for comparison and depend heavily on the total number of points in the distribution. The standard deviation of the means σ(μ) is the best measure for comparison and is highly consistent; however, it is also an absolute measure, meaning if the baseline changes in μ were orders of magnitude greater or smaller, the measured values would also be equivalently change. This makes this measure hard to interpret or use to detect anomalies. Meanwhile, for continuous distributions, the Jensen–Shannon divergence and incoherence increase rapidly initially but give consistent measures based on the relative difference between the μ of the differed distribution to the others.

The middle row (B) of Figure 7 has 12 Guassian distributions μ=1,σ=0.1 plus a single distribution with μ=1,σ=0.1+x. Here, σ(σ) has a similar detecting effect to σ(μ) above. But, it has the same downsides of being absolute. Incoherence and Earth mover’s distance are the only measures that are able to detect the difference in this skew. ANOVA and Kruskal tests, meanwhile, are effectively random, as seen with the large error bars. This is because they assume homoscedasticity (i.e., the values require the distributions to have the same variance). Finally, we see that since $D_{JS}$ does not have the squared term (does not use the second moment), it is unable to detect these changes.

The bottom graph (C) of Figure 7 looks at how the values change over the full range. Here, we compare three uniform distributions of U(0,x), U(0.5−x/2,0.5+x/2), U(1−x,1), so that when x=0, this approximates an identity matrix where the distributions are at their most inconsistent. Meanwhile, when x=1, the three distributions are all identical uniform distributions across U(0,1). It should be noted that the continuous entropy estimator used can compare values over any set of values, but throughout these trials, we normalize all inputs to between [0,1]. Here, we see again that the Earth mover’s distance does a good job at quantifying the consistency, but is hard to put those values into context based on a comparison.

**Figure 8:**
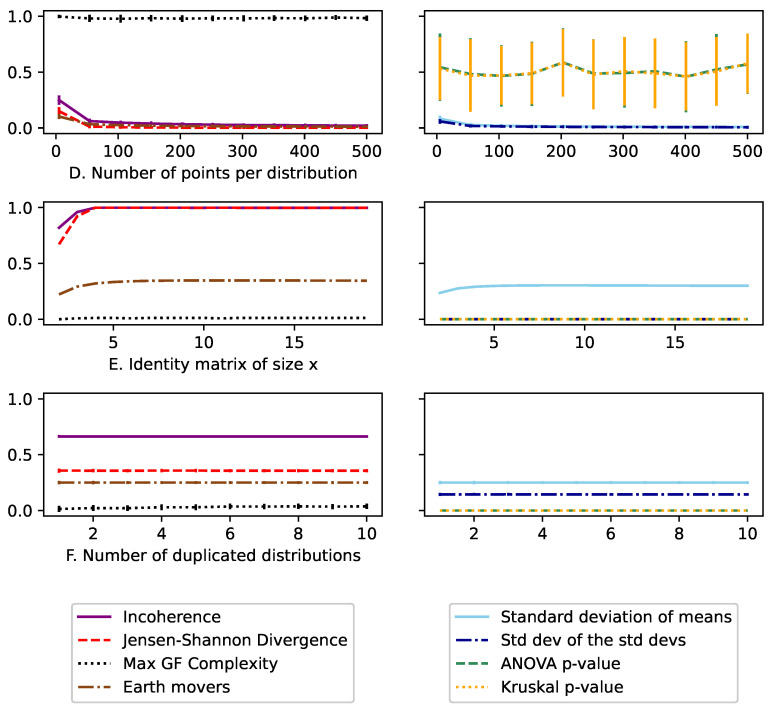
In this Figure, we look at cases where incoherence should be consistent. The **top row** shows that low volumes of points taken from a random distribution cause incoherence to rise. The **middle row** shows how incoherence is bounded by 1 when approximating an identity matrix in a continuous format. However, this bound is lower when analyzing too few distributions, as this approximation breaks down. Meanwhile, the **bottom row** shows how consistent the values are, even with increasing numbers of distributions. Also, the value of incoherence is the same as in its equivalent discrete case in Figure 5, showing the consistency of the measure across discrete and continuous cases.

Figure 8 looks at cases where we should see consistency in the results. The top row (D) compares 20 Guassian distributions with μ=1,σ=0.2 with *x* points selected from those distributions. Since the points are selected randomly, we see how at low volumes, these random distributions appear to be more inconsistent and therefore incoherent. It is important to note how high incoherence trends at low observation counts due to the active bias of wanting to be pessimistic.

The middle row (E) of Figure 8 then looks at approximating an identity matrix of size *x*. This was done using *x* distributions, where each individual distribution had all the values set to i/x. The first major thing to note is that we cannot properly approximate an identity matrix with continuous variables with low numbers of distributions. This is because when the pooled distribution is created, there is still be only a few clear peaks. It is only at a higher *x* value that these peaks would begin to smooth into a uniform distribution. This means that incoherence is bounded at a lower value when using fewer distributions, which practically means that we should ensure we are always sampling with more than six distributions in an ensemble.

Finally, the bottom graph (C) of Figure 8 compares *x* numbers of identical distributions; specifically, *x* copies of a minimal entropy distribution (all values are 1) against a maximal one (a uniform distribution U(0,1)). Here, we see that all the measures are invariant, because the inconsistency is not changing. But, we also see that the value of incoherence is I=0.65, which is the same as the discrete case for minimum vs. maximum entropy states in Figure 5.

### 5.2. Features of Complexity

#### Disorder and Order

As has been discussed, complexity can be seen as balance between order and disorder. As mentioned in the introduction, at first glance, this description certainly fits but is arguably incomplete. One would not typically consider the dispersion of an ideal gas to be a complex system because it merely evolves from a low to high entropy state. Here, we propose a more nuanced distinction, where the order in each sample (instance of the system) must be distinguishably different from the other samples.

**Figure 9:**
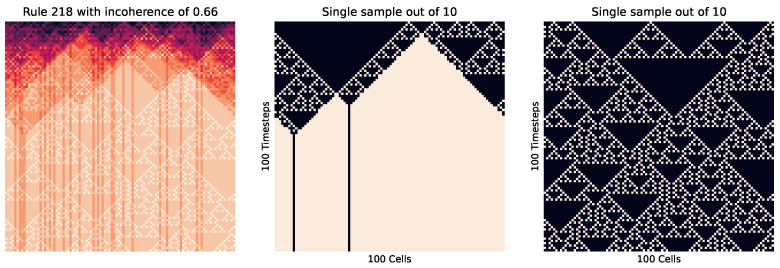
Rule 218 cellular automata. There is a heatmap of all 10 trial results on the left, with two of those individual trials to the right. This rule is wolfram class 2, which is typically considered not complex. However, the wolfram classes look at the state of an individual sample as it evolves over time (i.e., comparing rows of cells downward), whereas here, we have been comparing the state across time (the entire 2D grid) against other samples. For the other examples (rules 220, 0, and 30), there is a high correlation between these two perspectives. However, this rule highlights where these views diverge and the versatility of incoherence. For instance, we can calculate the incoherence from the wolfram perspective by treating downward rows as an ensemble of trials. In this case, we find that in most trials, there is a low incoherence, since over time, the patterns are the same and highly predictable. This means that there *can* be a strong agreement with the wolfram classes and incoherence. However, taking an ensemble perspective over multiple samples, we can see that each trial is highly sensitive to initial conditions and behaves very differently, to the extreme in the difference between the central and right graphs, leading to a high incoherence of 0.59 and an unpredictability that was not measured based on previous analysis.

First, we look at the cellular automata in Figure 9, which visualizes this concept. Here, we see on the right how instances of the same rule (with differing starting cells) can produce wildly different patterns, where each of those patterns are of a relatively low entropy, while the entropy of the pooled distribution (on the left) is much higher.

**Figure 10:**
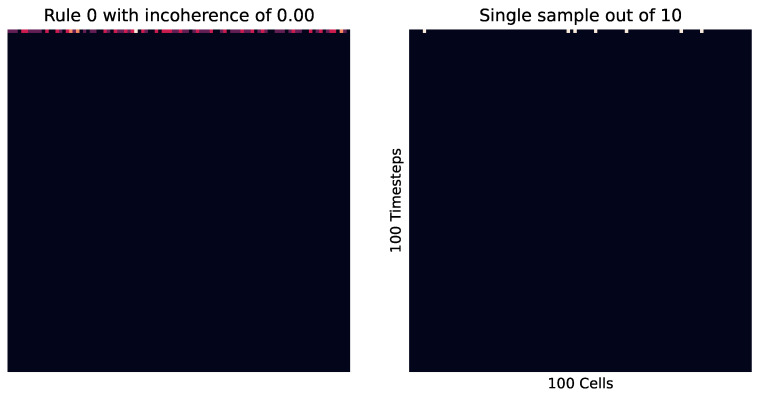
Rule 0 cellular automata. This is wolfram class 1, where the results are highly ordered and uniform. We see that no matter the initial state of the cells, every iteration leads to the same outcome, meaning this is very coherent and ordered, with an incoherence value of 0.00.

**Figure 11:**
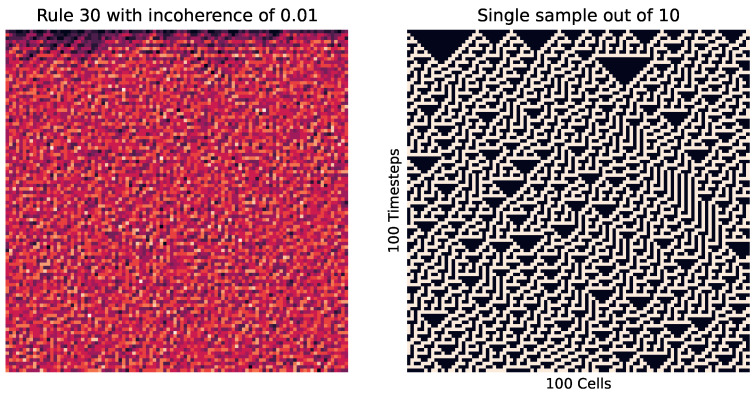
Rule 30 cellular automata. This is wolfram class 3, where the results are highly disordered and unpredictable; in this case, creating a high level of entropy. Here, each iteration is not exactly the same. However, there is no clear distinct or ordered pattern, leading to a low incoherence value of 0.01.

Meanwhile, Figure 10 shows how in a highly ordered system, which results in the same outcome every time (no matter the initial cell placement), results in a small incoherence. Meanwhile, Figure 11 shows the reverse, with a highly disordered (and high entropy) rule. At these extremes, the order and disorder is so great that no matter the initial setup, the result across every instance is the same.

**Figure 12:**
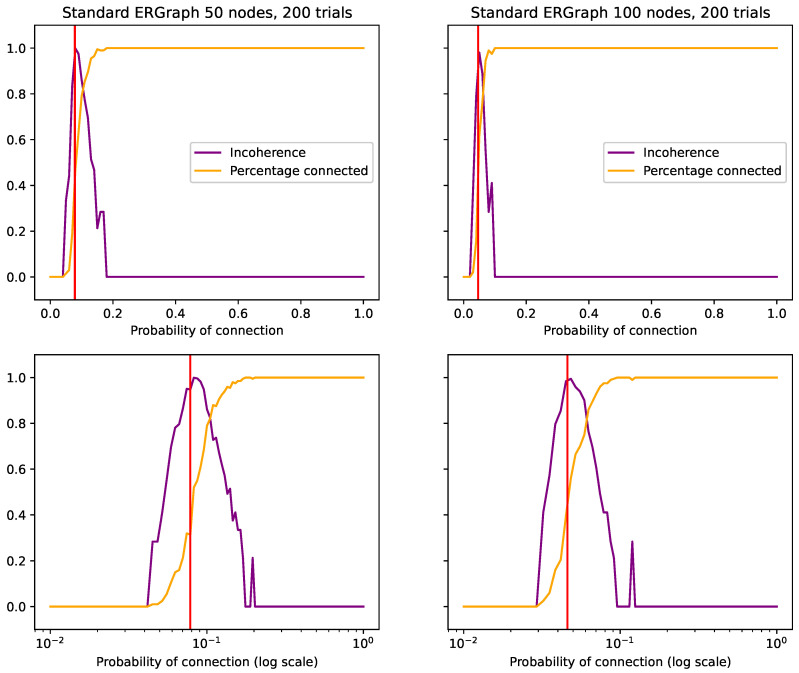
The vertical red line is the theoretically determined critical point of p=ln(n)/n for an *n* node graph. The left hand side is for n=20 and the right is n=100. We see that in both cases, incoherence largely agrees with this theoretical tipping point, but it does have a spread of values that correlate with the percentage of graphs that were found to be connected. Interestingly, although incoherence is close to the theoretical tipping point, its maximal value is slightly higher when the percentage connected is 0.5 (dashed red horizontal line).

Next, we look at this using Erdos–Renyi graphs. These are well studied as a balance of order and disorder. When p=0, the probability of connection is 0, and so no edges can be made. Therefore, every graph is identical. Meanwhile, when p=1, every connection is made, and so again, every graph is identical. Meanwhile, when p=0.5, the probability of connection is at its most disordered, and the number of connections per node is at its highest entropy. However, each instance of these graphs presents an overall identical structure, where the distribution of counts across nodes is identical. This is why Erdos–Renyi graphs are studied, because from their disorder comes remarkably predictable patterns such as those shown in Figure 12.

**Figure 13:**
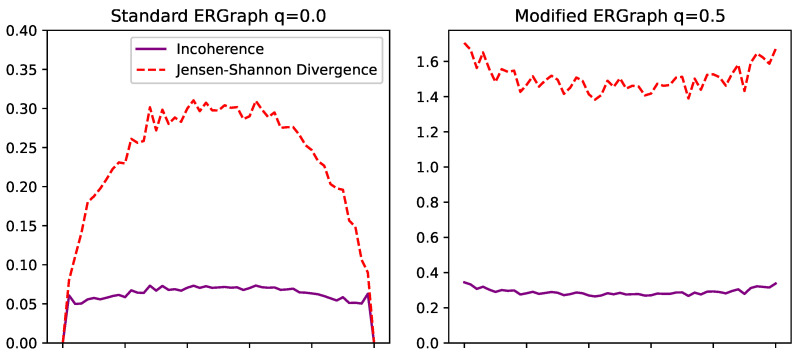

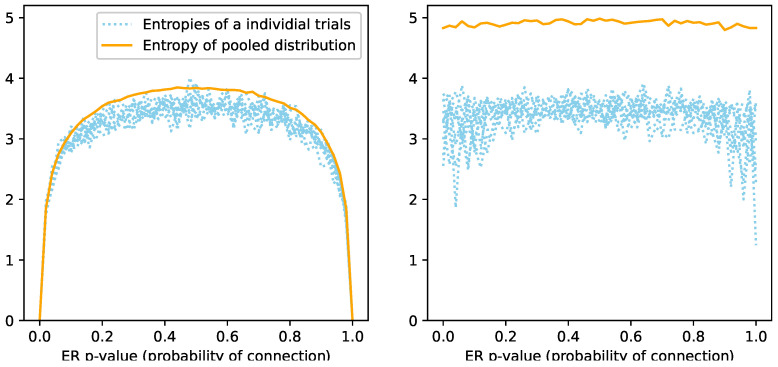
An ER graph with 50 nodes and 100 trials compared at multiple values of *p*. On the left, we look at standard ER graphs, while on the right, we compare this to our modified version. On the **top left**, we see how since the Jensen–Shannon divergence is an absolute measure and relative to the pooled entropy, it follows the curve of the pooled entropy. Meanwhile, incoherence is approximately flat across the entire spread I≈0.07, recording a low inconsistency from the minor randomness from each trial, similar to an idea gas, dropping only to zero when p=0|1, where the graphs are truly identical. On the **right**, we can see where the *p* value is slightly randomized on a per instance (system) level. Here, we see how incoherence is markedly higher at I≈0.28. In the **bottom right**, we now see how the entropy of the individual trials is much lower than the pooled entropy compared to the standard case (shown in the **bottom left**).

Figure 13 shows this with a 50 node graph, compared 100 times. Here, we see the standard graph on the left, following a curved symmetrical path as it progresses from the lowest entropy to the highest, then back to the lowest again. In the bottom left graph, the entropy of the pooled distribution is remarkably similar to the distributions of each of the individual trials, meaning they are very similar.

On the right, we introduce some disorder on the system level. On the bottom right, we see now how although each of the individual entropies is similar to the standard case, each instance is clearly very different, as the pooled entropy has been driven to be much higher, leading to a higher recorded incoherence.

It is also worth noting in the top left graph that the Jensen–Shannon divergence follows a curved path, while incoherence is consistently low. This is because incoherence is normalized by the pooled entropy, allowing it to be a more robust measure.

Therefore, incoherence does not measure the balance of order and disorder on an instance level. Instead, it measures when a system is ordered on an instance level but disordered at a structural (model) level.

### 5.3. Criticality

Criticality is typified by a sudden change in a system, be it ice-sheet collapse or a state change from liquid to gas. It is commonly seen as a feature of complexity [8,9].

Erdos–Renyi graphs are considered “connected” if they can be traversed from any node to any other via only edge connections. These graphs have been shown to have a critical point, where an *n* node graph will suddenly go from being disconnected to connected at the threshold p=ln(n)/n.

In Figure 12, we compared graphs where the state of the graph was [1,0] if the graph was disconnected and [0,1] if it was connected. What we see is that incoherence peaks around the theoretical tipping point. However, we also see that incoherence also quantifies how sudden the tipping point is (via the spread). Where we see the spread of values n=20 is markedly larger than when n=100. In this case, the spread and incoherence is correlated to the percentage of graphs that were connected for *p*. What is interesting is that at the theoretical tipping point, the percentage of connected graphs is ≈0.4, while when incoherence is maximal, the percentage is closer to ≈0.5. This highlights how hard it is to quantify a specific singular value of any tipping point and how incoherence offers an ability to quantify that spread.

**Figure 14:**
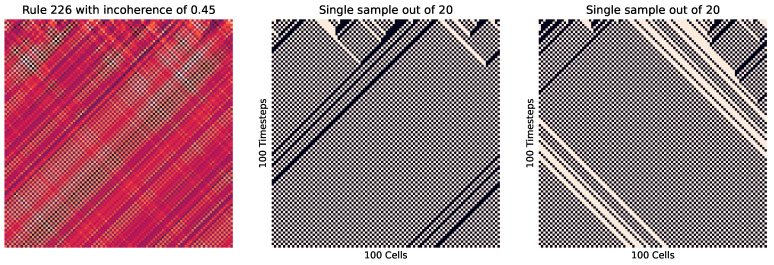
Cellular automata rule 226 with $p=0.\bar{50}$. Here, we see there is a critical point that causes the higher central incoherence values in Figure 16, such that depending on the initial conditions (of this entirely deterministic ruleset), some samples create diagonals (**left**) and other patterns (**right**). The left hand graph shows a heatmap of all the samples pooled together (creating a crosshatch), while the **central** and **right** graphs show single samples with this specific *p* value striping in opposite directions depending on the initial state.

**Figure 15:**
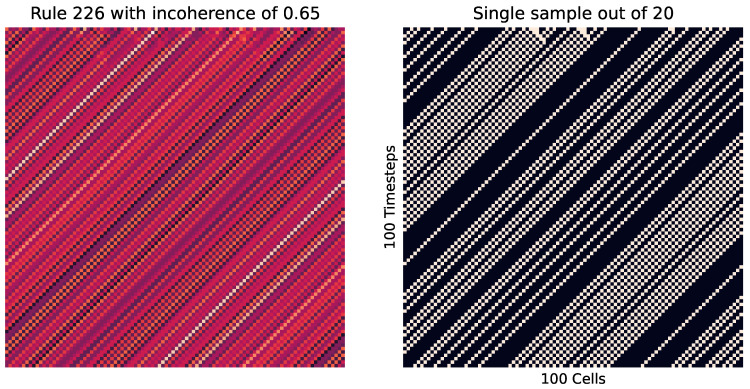
Cellular automata rule 226, with p=0.73. We see that this incoherent rule creates an interesting stripe patterns, which are unique per sample (hence why it is has a large incoherence value) but are uniform in direction. For p<0.4, the pattern is reversed.

We can also see how incoherence helps identify criticality in cellular automata. We do this by changing the percentage *p* of 0 values to 1 for various rules. Looking at rule 226 in Figure 14 at p≈0.5, we see how in some instances there is a striping left, while in some there is a striping right. Meanwhile, at higher or lower values (as seen in Figure 15), the striping is consistent.

**Figure 16:**
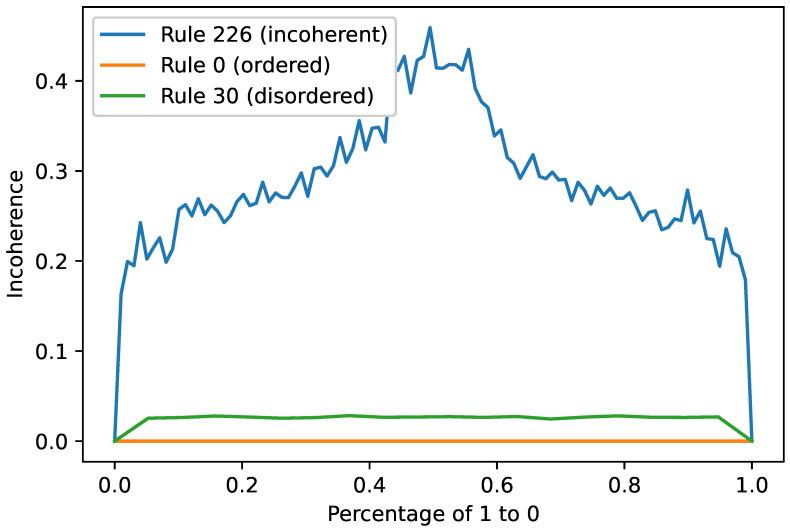
Cellular automata rule 226 tests two types of sensitivity to initial conditions on cellular automata for incoherence *I*. Firstly, *p* represents the proportion of 1 cells to 0 starting cells. Secondly, for each of the 20 samples created at each *p* increment, the placement of those 1 cells was random. What we first see is that sensitivity to the initial condition of placement only applies to the complex case (rule 226, blue) and not the ordered (rule 0, orange) or disordered (rule 30, green) cases. What we also see is that this sensitivity for the complex case is variable depending on the initial proportion *p*, meaning that incoherence is a parameter that is dependent and not a feature of an overall system per se.

Figure 16 firstly shows that incoherence is not only a measure of the system but also a measure for the given set of parameters. Here, we see that the striping pattern (as shown in Figure 15) is incoherent, as there is some variability between instances. But, the variability is far less than at the peak around p=0.5 where the tipping point is. We also see that some other rules (such as 0 and 30) do not measure any sizable incoherence, no matter the value of *p*.

**Figure 17:**
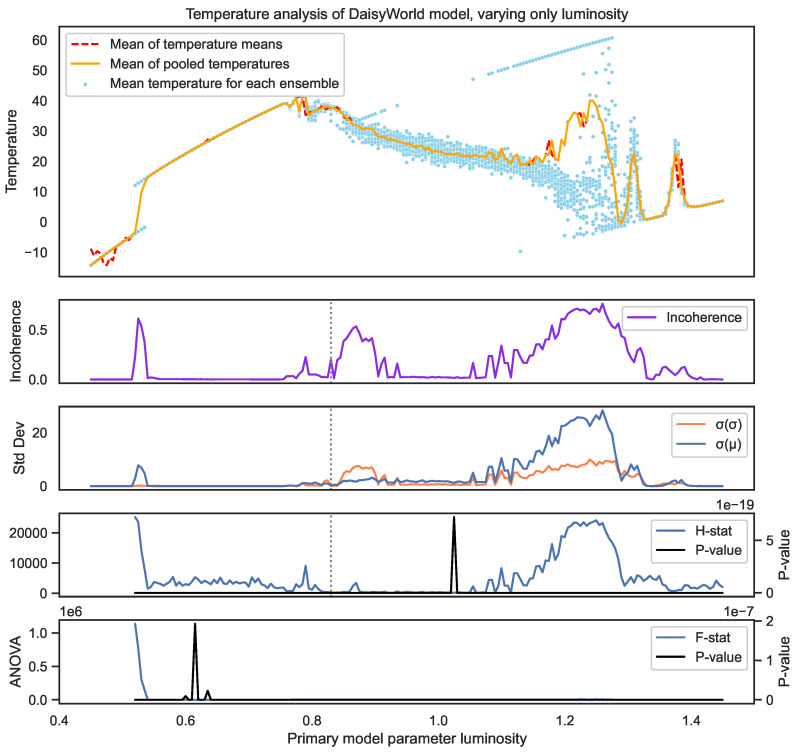
Daisy world model. The x-axis varies the luminosity of the sun in the world. For each luminosity value, the simulation was run 20 times, varying just the initial positions of the daisies randomly. Here, we are looking at the distribution of temperatures across the world at the final step. In the top graph, the blue dots represent the mean global temperature for each trial. The dashed red line is the mean of the blue dots (i.e., the mean of means). The orange line is the mean of the pooled distribution. The second graph shows the incoherence of the temperature distributions. The third graph shows the standard deviation of the mean and the standard deviation of the standard deviations for each trial distribution. The fourth shows the Kruskal H and *p*-values, while the bottom graph shows the ANOVA F and *p*-values. There are a few notable things that this graph illustrates in a reasonably sophisticated use case. The first thing we notice is how the Kruskal *p*-value and ANOVA values fail to offer any useful information in this case. For the ANOVA, this is because it assumes consistent variance across test distributions, which is a poor assumption in real-world situations or even for mildly complex scenarios. The next thing we see is how incoherence picks out phase changes in the system. For instance, around a luminosity value of 0.55, we see how trials start to fall into two predictable scenarios, where the system goes from where no daises can survive to some black ones surviving. Kruskal H and the standard deviations both also detect these points of criticality. Incoherence, however, is the only measure that accurately identifies all the parameter values that lead to inconsistent results. Moreover, although the standard deviations and Kruskal H-statistic are able to identify some of the inconsistencies, their actual values are relative the temperature values and are hard to interpret against other systems.

Next, the Daisyworld model is a system that is typically thought of as being highly self-organizing. In this model, as seen in Figure 17, there are three tipping points. The first occurrs when the system goes from always being dead to where some black daisies survive at a luminiosity of l≈0.55. Then, around l≈0.9, some white daises also survive. Then, around l≈1.25, the system destabilizes back into death.

What we see in this case is how different the criticality is in these cases. The left-most tipping point at l≈0.55 falls into two clearly different scenarios, while the right-most breaks down much more slowly and into a wide array of different eventual scenarios.

**Figure 18:**
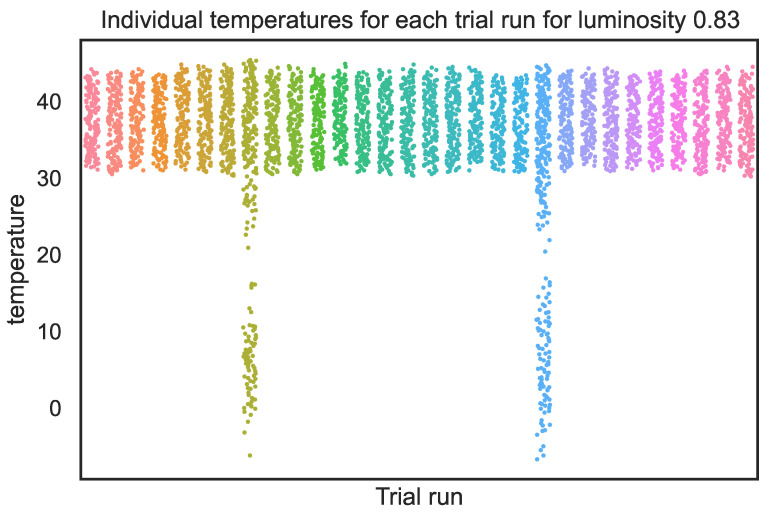
Temperature distributions for each of the twenty trials at a luminosity of 0.83 for the Daisyworld model. The x-axes are categorical, showing each trial run in a different color. The added x-axis scatter within each trial is present so that each point can be seen more easily. This figure is highlighted in Figure 17 by a vertical grey dotted line. We single out this particular instance, as it demonstrates a unique difference in incoherence against the other standard continuous statistic tests. ANOVA, Kruskal, and the standard deviations tests do not see these distributions as inconsistent because only two out of the twenty are outliers. Incoherence is designed to overweight the significance of outliers. In this specific case, these distributions show varying long tails, which are typically poorly identified using standard techniques yet are a common occurrence in complex systems.

We also see that ANOVA and Kruskal are poor measures for consistently identifying these tipping points. The standard deviation measures of the mean σ(μ) and standard deviation σ(σ) are useful but not comprehensive, such as at l=0.83 (shown as a vertical dotted line) and the point to the left at l=0.80. Figure 18 shows the actual distributions for l=0.83. We see that although most of the distributions are consistent, two have long tails. This makes them hard to detect because their mean and standard deviations are largely consistent.

In all cases, incoherence accurately spots any type of inconsistency in the result, whereas relying on any other single metric fails to do so.

#### Sensitivity to Initial Conditions

“A dynamical system is called ‘stable’ if it reaches the same equilibrium state under different initial conditions” [8].

This feature is typically measured using Lyapunov exponents. However, this technique requires knowing the exact analytical form and equations for the dynamics of the system, such as a double pendulum. However, many stochastic systems cannot be expressed this in form, and this is the reason why the method of using agent-based models was created. Unfortunately, this means that Lyapunov exponents cannot be applied in many contexts. Incoherence instead relies on the distributions and the output data that characterize the system rather any input equations.

We have already seen how incoherence can be applied in this way for cellular automata. Figure 9 and Figure 16 involved a sensitivity to initial conditions, where the macro conditions were kept consistent (with a consistent 0 to 1 percentage, rule, and cell count) but the micro conditions of which cells were in which binary state were varied on an trial-by-trial case. But, we have also seen how incoherence is not limited to this, since in the standard Erdos–Renyi graphs, the randomness was not an initial condition but rather a part of the process of creation.

### 5.4. Perturbation

“A system might be robust against perturbation in the sense of maintaining its structure or its function upon perturbation” [8]. This can also be called ‘stability’ or ‘resilience’.

Perturbation is most commonly measured by looking at the time it takes to return to its previous structure. Although incoherence can measure this by comparing the before distribution to the distribution at time *t* after it is perturbed, there are other measures that can do this. This also assumes that given the same parameters, that system is coherent, and we would measure the same results under every trial of this experiment. Incoherence can give a measure of stability across trials, similar to criticality, where we are measuring what proportion of worlds the system returns to their previous structure (and how different are those new states).

We explored this using the Daisyworld model by introducing a plague to kill *p* percent of the uniform daisies randomly. We could then measure how robust the system was in terms of how much each sample would settle to the same distribution as each other. This is important, since the Daisyworld is a highly self-organized system and is often considered to be coherent for all parameter values. However, what we discovered was that perturbation often resulted in a loss of this coherence, and incoherence was an effective measure of this.

### 5.5. Diversity of Diversity

“The term ‘diversity’ is often used to describe inhomogeneity in element types…The simplest measure of diversity is the number of types or the logarithm of that number. A more informative measure takes into account the frequency of each type…Treating such frequencies as probabilities, the *Shannon entropy* H(X) is used as a measure of type diversity. In ecology, diversity is measured using the *entropy power*$2^{H}\left(X\right)$ or $e^{H}\left(X\right)$” [8].

Again, incoherence, *I* can be used to measure a variant of diversity; specifically, the ‘diversity of diversity’ *across* systems, where we look at how different the distributions of ‘element type’ vary are across different samples.

Using the Daisyworld model, we found that when we introduced a higher diversity into the samples where there were three types of white daises and three types of black daisies, each with a different albedo, what we found was that the incoherence *I* massively dropped. This is because although the available element types increases, thus increasing plain ‘diversity’, the critical point at which was there for only two species types was smoothed out, importantly causing a higher coherence between samples, meaning that *I* was lowered, as each sample found the same equilibrium point. This means that the ‘diversity of diversity’ is lower for this point, despite the ‘diversity’ being higher. While we also found was that when the samples were allowed to mutate, each sample ended up having a different species that was particular to that specific trial. Thus, increasing *I*, representing a higher ‘diversity of diversity’, now also results in a higher ‘diversity’.

## 6. Motivated Use Case—Real-World Decision Making

To demonstrate how incoherence can be useful, we shall look at a real-world use case. In this example, imagine you own a silicon chip manufacturing plant. In order to produce a chip, it needs to undergo a series of processes, such as photolithography [41]. For each process, you have a set of machines that can do that process. You commission a digital twin of the factory to simulate the expected effectiveness of different upgrade options.

When constructing this digital twin, it would be easy to assume the results are not incoherent and are ergodic; i.e., the result of any single simulation is representative of any other simulation with the same parameters. However, this factory can be thought of as a network, which notoriously features complex dynamics [42,43,44] and is therefore likely to be incoherent.

**Figure 19:**
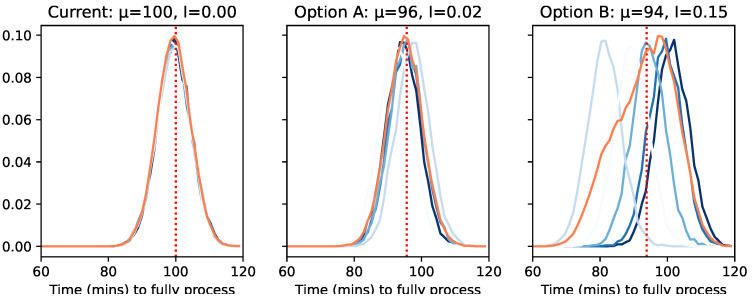
Illustrative results for visualizing the idealized use case. These represent the results of a digital twin of a manufacturing plant. The distributions represent the steady-state times it takes for goods to be produced end-to-end. Each graph shows the individual probability density functions of five trial simulations in blue. The pooled distribution is shown in orange. The title displays the value of the pooled mean μ (dashed red line) and the Incoherence *I*. The idealized use case section (Section 6) describes how to interpret these values in the context of real-world limitations.

The current setup has six types of machines that are organized into layers. The system today is coherent but relatively inefficient, as shown in Figure 19. The figure also shows the distributions of two potential upgrades: option A, which re-arranges the physical layout of the machines in the building, and option B, which introduces a queuing system to compensate for the physical distance between the machines.

Option B best demonstrates the risk of assuming a system is coherent. If you had run the digital twin only once, at the extremes, you might have thought this upgrade was unduly positive (left-most distribution μ=82) or negative (right-most, μ=102). This means that when it was implemented, you as the factory owner would quickly jump to the conclusion that the digital twin is inaccurate at best or misleading at worst, blaming those who created it. What incoherence can bring is a measure of certainty about how likely a perfect model is to be able to represent the single instance of reality.

In many cases, analysts might be tempted to run multiple simulations and use the pooled distribution (point-wise mean shown in orange) as a good proxy of what is likely to happen in reality. However, this too can be very misleading.

For instance, running a factory requires huge amounts of external scheduling of staff and raw materials. This means that incorrectly predicting the variance of a distribution in these cases means also incorrectly predicting the daily volume or over-estimating when you are likely to obtain the first output for quality assurance purposes. This means that it becomes very possible that option A is now more appealing, despite offering a much smaller improvement. On the other hand, if it is easy and cheap to restart the system, it could make more sense to go for option B, as you can restart the system until you find a more appealing instance.

Here, we have directly visualized the distributions of each trial to give an intuitive sense of the trade-offs. However, in real-world cases (such as the Daisyworld example in Figure 17), the level of incoherence varies based on the values of the parameters (e.g., the max length of the queue or the number of multi-process machines), making it nearly impossible to visualize these specific trade-offs if you have more than a single parameter. This makes a single number important for automatic identification.

Traditional statistical tests (such as ANOVA or chi-squared) are optimized around deciding *if* the distributions are the same, not by *how* much. Even then, they are poor at identifying systems with occasional outliers. Similarly, many statistical tests have other limitations, such as assuming consistent variance across distributions or assuming that the distributions are Guassian in shape. Most commonly, they can only compare two rather than N distributions.

This use case was designed to highlight the importance in decision making. Structurally, this use case is similar to many real-world examples, such as parcels through a supply chain or packets through micro-services. Also, although in this case, the incoherence was driven by complex dynamics in the system, the measure of incoherence could be used when evaluating an ensemble of climate models using different equations; for instance, giving a measure of model agreement.

## 7. Discussion

### 7.1. Ergodicity

In the broadest sense, ergodicity [11] and incoherence are consistent in their interpretation: we cannot use a single sample distribution to accurately represent the ensemble, no matter the time period it is sampled over. Also, they are both a feature of a system and not the data about a system.

However, they have some fundamental differences. First, ergodicity is a binary measure, whereas incoherence is a continuous quantification.

Secondly, ergodicity is defined as follows: for a stochastic process or dynamical system, if a variable of that system uniformly and randomly visits all parts of the state space, that system is called ergodic. This means that the typical behavior of a system can be inferred from the trajectory of any point. Informally, this implies the time average of a variable matches the ensemble average. Therefore, non-ergodic systems are inherently incoherent, but incoherence can be used to measure more than ergodicity, since although we could create an ensemble of distributions over time, the distributions could represent the populations.

### 7.2. Existing Non-Parametric Statistical Tests

Statistical tests of homogeneity give a measure of how confident one can be that a set of sample distributions comes from the same underlying distribution. Therefore, contrary to the name, they assume that they are the same by default, but with sufficient evidence, we can say that the distributions are different.

Therefore, independence tests measure how confident we are about the differences in distributions. Incoherence is designed to be an accurate and robust measure quantifying a feature of the system, not providing information regarding the confidence of the result. Therefore, incoherence is invariant to increasing evidence, except to gain accuracy. This is due to it being analogous to variance, where it is designed so that we can have a meaningful conversation about the level of incoherence of an ensemble rather than how confident we are in the binary measure of it being incoherent. More formally, incoherence is a measure of the irreducible [7] aleatoric uncertainty caused by the heterogeneity of the distributions, while independence tests combine this with the epistemic uncertainty of whether a system is heterogeneous due to by having too few observations.

Secondly, incoherence is non-parametric, in the sense that it does not require the distributions to be normal or have constant variance (homoscedasticity). In fact, what is more, it can detect differences in variance as well as just means while also detecting differences in distribution shapes.

Thirdly, since the metric is based on the entropy of a system, we no longer have to pick the right measure for the given data and model. Instead, we have a consistent measure, but we tailor how to manage the inputted variables. This means that incoherence can work with both continuous and discrete variables (using the appropriate entropy estimators). It can deal with any number of sampled distributions (often called categories), unlike most *t*-tests, which allow for only two. It can deal with any number of output variables, unlike any other non-parametric measure which only allow for one, enabling it to be a consistent and comparable measure across nearly any model or situation and allowing for a consistent terminology and baseline for policymakers.

### 7.3. Requirement of Multiple Samples per Ensemble

This technique requires the existence of multiple measurable systems to create an ensemble. Unfortunately, many of the complex systems we interact with outside of simulations (such as the global economy or our biosphere) have only one accessible instance. In this case, the technique can be adapted to answer the question: what sub-systems within the wider system can we consider to be coherent samples of each other? The hierarchical nature of complexity is another acknowledged feature of it [8] that could be a formal definition of hierarchical complexity proposed by Herbet Simon.

## 8. Conclusions

Here, we have established the value of a new measure, *incoherence*, with three key practical uses.

Firstly it has a clear and intuitive explanation for non-statisticians to understand a perspective of complexity, where incoherence does not measure the balance of order and disorder on an instance level. Instead, it measures when a system is ordered on an instance level but disordered at a structural level.

Secondly, we demonstrate that it has a clear value in real-world decision making.

Third, we show how it can be used as a *standardized* measure of ensemble divergence (internal variability) across many different ensemble types, including multi-model (structural) ensembles that compare many different physical models and proxy equations. Perturbed physics ensembles investigate differing macro parameters to within their known uncertainty. The initial condition ensembles can inform policy decisions (forcing). There are many more potential applications in machine learning, such as ensuring that the distributions of bagging from bootstrapped data are consistent, or stacking prediction confidence [45].

Furthermore, each specific model beyond these ensemble types will vary in terms of the number and type (continuous or discrete) of output variables. The benefit that incoherence brings is that since the input entropy measure can be tailored to the model, it can be used robustly and consistently across all models and ensembles, offering an intuitive way for model builders and policymakers to establish baselines regarding the inherent aleatoric uncertainty in their models.

Fourth, we showed that this measure correlates with a number of features of complexity. In particular, it specifies a balance between order and disorder, sensitivity to initial conditions, criticality, perturbation, and diversity.

Finally, it is a double omnibus measure, meaning that it not only works to identify if the ensemble as *a whole* but is also incoherent (rather than identifying the samples that deviate), meaning it is like a non-parametric test that identifies if there are *any* differences in distributions. This means that it can identify differences in means (like an ANOVA) and also variances (unlike an ANOVA). Like above, it can do this for any number of distributions and any number of output variables (unlike any other continuous non-parametric test). Having said this, it is not a test of independence, and it should not be seen as a replacement but rather as a compliment. 

## Data Availability

The datasets generated and/or analyzed during the current study are available in the incoherence repository: https://github.com/timjdavey/Incoherence, accessed on 4 June 2024.
